# Present Focus Moderated the Relationship Between Academic Stress, Self‐Stigma, and Anxiety Level: A Longitudinal Study

**DOI:** 10.1155/da/7831491

**Published:** 2026-04-24

**Authors:** Minqi Yang, Shuai Luo, Miao Miao, Peng Han, Linxi Yan, Guofang Wang

**Affiliations:** ^1^ School of Education, Zhengzhou University, Zhengzhou, 450001, China, zzu.edu.cn; ^2^ School of Sociology, China University of Political Science and Law, Beijing, 100088, China, cupl.edu.cn; ^3^ Department of Medical Psychology, School of Health Humanities, Peking University, Beijing, 100871, China, pku.edu.cn; ^4^ Office of Student Affairs, Beijing Youth Politics College, Beijing, 100102, China

**Keywords:** academic stress, anxiety, learning difficulties, present focus, self-stigma

## Abstract

The study was mainly aimed to explore reciprocally temporal relationships between academic stress, self‐stigma, and anxiety level, and the moderating effect of present focus among high school students with learning difficulties (LDs). Academic stress, self‐stigma, anxiety level, and present focus were measured at Time 1 (T1) and Time 2 (T2, 5 weeks later) among 3000 senior high school students in Liaoning, China. Using structural equation modeling (SEM), the researchers conducted cross‐lag analyses of academic stress, self‐stigma, and anxiety level among two samples of the selected 735 students with LDs (M ± SD = 16.32 ± 0.97 [years]) and 733 students with excellent performance, respectively. Correlation analysis showed that the positively simultaneous and successive correlations between academic stress, self‐stigma, and anxiety level of the students with LDs were significant. Cross‐lagged analysis showed that, among the students with LDs, academic stress at T1 only had a positive relationship with academic stress at T2 (*β* = 0.36, *p* < 0.001), whereas self‐stigma at T1 had positively predictive links to self‐stigma (*β* = 0.42, *p* < 0.001) and anxiety level (*β* = 0.08, *p* = 0.031) at T2, and anxiety level at T1 had positively predictive links to anxiety level (*β* = 0.37, *p* < 0.001), academic stress (*β* = 0.13, *p* = 0.001), and self‐stigma (*β* = 0.10, *p* = 0.006) at T2. Whereas among the students with excellent performance, academic stress at T1 was positively related to academic stress (*β* = 0.39, *p* < 0.001) and anxiety level (*β* = 0.09, *p* = 0.015) at T2, anxiety level at T1 was also positively linked to anxiety level (*β* = 0.35, *p* < 0.001), academic stress (*β* = 0.13, *p* < 0.001), and self‐stigma (*β* = 0.10, *p* = 0.026) at T2, whereas self‐stigma at T1 only had a reciprocal relationship with itself (*β* = 0.49, *p* < 0.001) at T2. Besides, high present focus at T2 exaggerated the temporal association of academic stress at T1 with anxiety level at T2 among students with LDs (*β* = 0.01, *t* = 2.07, *p* = 0.039, 95% CI [0.001, 0.024]). This investigation elucidated the dynamic patterns of anxiety level, offering a nuanced comprehension of its relationship with academic stress and self‐stigma, and the moderating role of present focus. Moreover, the results hold implications for the prevention and intervention targeting the reduction of anxiety among students with LDs and students with excellent performance.

## 1. Introduction

Students with learning difficulties (LDs) are, by definition, of normal intelligence and are free from sensory impairments but exhibit academic failure or significant underachievement compared with their peers [1, 2]. LD is different from specific learning disabilities (SpLD); those who are diagnosed with SpLD, their learning struggles are biological and permanent [3], but still can be high academic achievers [4]. LDs have been found to occur for various reasons among 12%–30% of students [5]. LDs can compromise students’ academic learning and motivation [6, 7], and are often common features of youth dropping out of school, overrepresented as juvenile delinquents, or experiencing mental health problems [8]. Besides, students with LDs have frequent struggles with schoolwork [9], which may increase their vulnerability to experiencing more negative emotions.

Anxiety of high school students refers to the negative emotional experience of high school students due to the threat to self‐value, which is caused by psychological conflicts and frustrations in the family and school [10]. Compared with students with excellent/very good performance, anxiety is significantly higher among the students with good/fair or pass academic performance [11]. Since anxiety has negative impacts on students’ academic achievement [12, 13] and health physically [14] and mentally [15], thus exploring the mechanism underlying the occurrence and development of the anxiety of students with LDs is of great value.

In China, academic excellence is highly valued. Students with the highest university entrance examination (Gaokao) scores enter elite universities and tend to receive the best job opportunities upon graduation [3, 16]. The phenomenon, described as by one exam determines one’s life, makes low academic achievers more likely to be targets of biased perceptions and generate self‐stigma [3].

Self‐stigma refers to the process of an individual accepting society’s negative evaluation and incorporating it into his or her own personal value system and sense of self [17]. Compared with students with academic excellence, students with LDs are also more likely to suffer stigma about their academic performance in China. Self‐stigma carries guilt and shame [18], which might compromise quality of life [19] and is associated with negative emotional reactions [20]. For instance, self‐stigma is associated with higher levels of psychological distress [17, 21], is positively associated with secondary traumatic stress [22], could predict depressive symptoms [23, 24], and is related to anxiety among mothers whose children were diagnosed with ASD [25].

Academic stress, defined as an adaptive psychological systematic process that occurs in learning environments when students face challenges and perceive them as stressful [26, 27], involves mental distress regarding anticipated academic challenges or failure or even an awareness of the possibility of academic failure [28], and has become a serious issue affecting nearly two thirds of senior high school students [29]. Compared with children, adolescents are confronted with more academic demands at school, and become more aware of individual differences in abilities and achievement [30]. Since attending a top university could bring about many more job opportunities in China [31], parents in China have high expectations of their child’s academic competence and ability to achieve [32], and often monitor and push them hard to achieve academic success [33]. Thus, these senior high school students are faced with greater academic stress than their Western counterparts [34].

According to the stress response theory, anxiety is a normal physical response to stress; thus, students exposed to a high academic stress environment may experience anxiety [35]. Empirical studies also showed that academic stress often leads to psychological and somatic distress [36, 37], is positively correlated with anxiety [38, 39], and is a risk factor for anxiety symptoms [40]. Besides, academic stress worsens the symptoms of depression and anxiety [26, 41]. In a longitudinal study, researchers tracked the stress and anxiety among students over the final year in seven Australian high schools, and found that stress and anxiety not only increased significantly but also were intracorrelated and correlated with each other over time [42].

Although both academic stress and self‐stigma were positively associated with anxiety level, not all students with LDs experience anxiety homogeneously, which suggests there are possible moderators. Personality generally augments or buffers the negative influence of the vulnerability factors on mental health [43, 44]. Temporal focus, as a stable personality trait, is defined as the extent to which people characteristically devote their attention to perceptions of the past, present, and future [45, 46], and often serves as a moderator of other relationships by either increasing or filtering out information, which depends on the time period(s) one typically considers [47]. Accordingly, having a strong “here and now” orientation could lead individuals to rely more on present‐day experiences and immediate surroundings, perceiving such information as more salient for attitudes and behaviors [48]. In the current study, the survey was conducted just after their latest academic examination scores (i.e., the current academic situations); thus, we explored the moderating effect of current focus (i.e., the tendency to devote an individual’s attention to thinking about the present [45]) on the links from academic stress to anxiety level. One study showed that students high in current temporal focus may be well aware of the risk situation and have the effects of temporal focus on emotions amplified [49].

Given that previous studies on LDs are mainly cross‐sectional, there is scant research about how the relationships among academic stress, self‐stigma, and anxiety level change over time among senior high school students with LDs and students with excellent performance from Asian cultures, especially in cultures that have a high focus on academic excellence, let alone comparing the differences in these relationships in students with LDs and those with excellent performance. Given that little is known about the possible moderating factors. Thus, the present study used a longitudinal survey design to investigate the reciprocally predictive relationship among academic stress, self‐stigma, and anxiety level, and to explore the moderating effect of present focus, in senior high school students with LDs and students with excellent performance, separately. Besides, we would explore the validation of the scales of the four constructs in students with LDs and excellent performance.

We hypothesized: (1) academic stress, anxiety level, and self‐stigma would be correlated with each other, and the correlation would be significant across time; (2) there would be temporal relationships between anxiety level and academic stress and self‐stigma; (3) a present focus would moderate the temporal links from academic stress and self‐stigma to anxiety level.

## 2. Methods

### 2.1. Procedure and Participants

Students in the randomly selected classes from two key high schools and three general high schools in Liaoning Province, China, were surveyed in two waves, with 5 weeks apart (since one person would experience state anxiety in a stressful situation [50], the 5‐week interval (the two waves of data collection were scheduled in the first and sixth weeks after the midterm exam) was selected to ensure the interval captures acute fluctuations without confounding effects from new stressors and to align with the local school’s academic schedule, since the semester in Chinese high school spans 18–20 weeks, midterms occur at weeks 8–10, and finals at weeks 17–20, with the 7–10 weeks between midterms and finals creating a natural stressor‐recovery cycle). The two tests were conducted in the classroom, with 3000 participants completing measurements at Time 1 (T1), and 2613 participants completing measurements at both T1 and Time 2 (T2). And then a total of 733 students with academic excellence and 735 students with academic difficulties were selected, respectively [51]. Students with LDs are those who possess intelligence within the normal range, but due to various reasons, are unable to adapt to learning within the context of regular school education, ultimately leading to “academic underachievement” or “poor academic performance,” which can be remediated given appropriate compensatory educational interventions. Specifically, those whose scores ranked in the bottom 10 of the class in the most recent examination (in the midterm or final academic examinations of the latest semester in the current study) were initially selected, while excluding students with intellectual disabilities or physical impairments. The initially selected group was then evaluated by their class teachers. Finally, students assessed by their class teachers as “students with LDs” were included as such in this study. Conversely, the operational definition for students with excellent performance is students whose scores ranked in the top 10 of the class in the most recent examination were selected [2]. The average age of the students with academic difficulties was 16.32 years (SD = 0.97; range = 14–19). The overview of the study’s three samples was shown in Table 1. To determine the required sample size, we conducted an a priori power analysis using 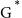
Power 3.1 [52]. Adopting a conservative approach, we specified a small effect size (*f* ^2^ = 0.02), *α* = 0.05, and power (1‐*β*) = 0.90. This yielded a required sample size of 713 participants per group. Our study included 736 students with LDs and 734 students with excellent performance, exceeding this threshold and confirming sufficient statistical power. All of the survey data were collected after informed consent was obtained from the participants. The university’s Institutional Review Board approved all procedures.

**Table 1:** The overview of the study’s samples.

Demographic variables	Total sample (*n* = 2613)	Students with LDs (*n* = 735)	Student with EP (*n* = 733)
	Frequency (%)	Frequency (%)	Frequency (%)
Gender			
Boy	1191 (45.6)	358 (48.7)	324 (44.2)
Girl	1422 (54.4)	377 (51.3)	409 (55.8)
Age			
14	63 (2.40)	10 (1.40)	21 (2.90)
15	546 (20.90)	138 (18.80)	169 (23.10)
16	998 (38.20)	283 (38.50)	279 (38.10)
17	751 (28.70)	219 (29.80)	203 (27.70)
18	243 (9.30)	80 (10.9)	58 (7.90)
19	11 (0.40)	5 (0.70)	2 (0.03)
20	1 (0.04)	0 (0.00)	1 (0.01)
High school grade			
Grade 1	1143 (43.7)	327 (44.5)	314 (44.0)
Grade 2	769 (29.4)	212 (28.8)	209 (29.3)
Grade 3	701 (26.8)	196 (26.7)	191 (26.8)
Residence			
Urban	986 (37.70)	273 (37.10)	261 (36.6)
Rural	1627 (62.30)	462 (62.90)	453 (63.4)
Only child			
Yes	918 (35.1)	260 (35.4)	243 (34.0)
No	1695 (64.9)	475 (64.6)	471 (66.0)

### 2.2. Measurement Instruments

Evaluation criteria for scale psychometrics were as follows: confirmatory factor analysis (CFA) with good model fit (CFI > 0.90, TLI > 0.90, RMSEA < 0.08, SRMR < 0.08; acceptable if CFI > 0.85, TLI > 0.85, RMSEA < 0.08, SRMR < 0.10) [53]; good internal consistency (Cronbach’s *α* ≥ 0.80, acceptable if ≥ 0.70) [54].

Participants’ academic stress was measured by the Students’ Academic Stressors Academic Stressors Questionnaire [55]. The scale consisted of five dimensions: task stress (17 items, e.g., too many tasks assigned by the teacher), competitive stress (9 items, e.g., competitors too strong), frustration stress (12 items, e.g., parents’ accusations), expectation stress (15 items, e.g., parents’ expectations), and development stress (3 items, e.g., talented but no opportunity to show). Each item was rated on a five‐point Likert scale (1 = no pressure, and 5 = a lot of pressure, almost unbearable). The score for each dimension is generated by adding the scores of items within the dimension, and higher scores indicate higher levels of stress. Based on an important qualitative research revealing the academic task stress is an important component of the stress of high school students [37], the present study applied the subscale of task stress with the *α* coefficient being 0.84 at T1, 0.89 at T2. CFA indicated good structural validity across the two time points (*χ* ^2^/*df* = 3.10, CFI = 0.90, TLI = 0.87, SRMR = 0.05, RMSEA = 0.05 at T1; *χ* ^2^/*df* = 3.79, CFI = 0.89, TLI = 0.86, SRMR = 0.056, RMSEA = 0.068 at T2) in the second‐order model of one higher‐order factor and three first‐order factors named work stress (five items), time stress (eight items), and required stress (four items). Factor loadings ranged from 0.42 to 0.82.

Self‐stigma was measured by the Internalized Stigma Scale for Middle School Students with Learning Disabilities, which has a 13‐items [56]. The scale includes three dimensions: the experience of discrimination (six items, e.g., Sometimes I feel that I am underestimated because of my poor academic performance), alienation (three items, e.g., If the academic performance is not good, students will not be willing to play with me), and concealment (four items, e.g., When the grade is not good, I try to avoid telling others). Each item was rated on a five‐point Likert scale (1 *=* strongly disagree and 5 *=* strongly agree). Higher scores indicate higher internalized stigma. In the present study, the *α* coefficients were 0.88 for total internalized stigma and 0.81, 0.79, and 0.66 for discrimination experience, alienation, and concealment, respectively. CFA indicated good structural validity across the two time points (*χ* ^2^/df = 3.39, CFI = 0.87, TLI = 0.85, SRMR = 0.053, RMSEA = 0.057 at T1; *χ* ^2^/*df* = 4.39, CFI = 0.91, TLI = 0.88, SRMR = 0.052, RMSEA = 0.068 at T2). Factor loadings ranged from 0.42 to 0.79.

The Chinese version of the state anxiety subscale of the State‐Trait Anxiety Inventory (STAI) Form‐Y, which was developed by Spielberger et al. [57] has good reliability, adequate concurrent and construct validity, and includes 20 items with 10 negative polarity items (e.g., I feel calm) representing the factor of state anxiety absent, and 10 positive polarity items (e.g., I am worried) representing the factor of state anxiety present [58]. Each item was rated on a four‐point Likert scale (1 = not at all and 4 = very obvious). The total score of the subscale was the sum of the 20 items, with higher scores indicative of higher levels of state anxiety. Based on the finding that the accurate assessment of state anxiety during before, during, and following evaluative events could be enhanced by using a reduced version of the S‐STAI that consists solely of positive polarity items [59], we applied the positive polarity items to measure the anxiety level in the current study. Because the factor loadings of Item 14 were 0.37 at T1 and 0.34 at T2 (less than 0.40), we deleted it in the CFA and structural equation modeling (SEM) in the current study. For the students with LDs, the CFA indicated good structural validity across the two time points (*χ* ^2^/*df* = 4.69, CFI = 0.95, TLI = 0.93, SRMR = 0.036, RMSEA = 0.071 at T1; *χ* ^2^/*df* = 3.85, CFI = 0.96, TLI = 0.94, SRMR = 0.032, RMSEA = 0.062 at T2) in the model of one factor (i.e., state anxiety present). The *α* coefficients for the factor were 0.88 at T1 and 0.88 at T2. Factor loadings ranged from 0.57 to 0.75.

Present focus was measured by the present focus subscale of the Temporal Focus Scale (TFS) [45]. The Chinese version of the scale has a good construct validity (the model fit in the confirmatory factor analysis: *χ* ^2^ = 67.50, *χ* ^2^/*df* = 1.32, CFI = 0.96, NFI = 0.89, RMSEA = 0.038) [60]. The subscale included four items (e.g., My mind is on the here and now). Each item was rated on a seven‐point scale describing the frequency with which the respondent thought about the time frame indicated by the item (1 = never; 3 = sometimes; 5 = frequently; 7 = constantly). Anchors for the TFS response scale were derived using guidelines from Bass, Cascio, and O’Connor [61]. The total score of the subscale was the sum of the four items, and higher scores indicate more tendency to focus on the present. In this study, the *α* coefficient of the subscale was 0.67. CFA indicated good structural validity across the two time points (*χ* ^2^/*df* = 4.524, CFI = 0.98, TLI = 0.88, SRMR = 0.019, RMSEA = 0.069 at T1; *χ* ^2^/*df* = 1.09, CFI = 0.99, TLI = 0.99, SRMR = 0.013, RMSEA = 0.011 at T2). Factor loadings ranged from 0.48 to 0.76.

### 2.3. Data Analysis

First, we used multiple imputation to deal with the missing data. The descriptive statistics and correlation analysis for all variables were conducted using SPSS (Version 25.0, IBM Corporation, Armonk, NY, USA). Then we evaluated multivariate normality using Mardia’s [62, 63] test. The results indicated severe nonnormality with both multivariate Skewness (*ps* < 0.05) and multivariate Kurtosis (*ps* < 0.05) for all the data for CFAs and SEMs in the current study (Table 2), necessitating the use of robust estimation methods. Accordingly, all CFAs and structural equation models were estimated using the MLR estimator in Mplus (Version 8.3).

**Table 2:** The Mardia coefficients of the variables in the present study.

Variables	Mardia Skewness		Mardia Kurtosis	
	Test statistic	*p*	Test statistic	*p*
Students with LDs				
AS T1	2069.58	<0.001	39.83	<0.001
AS T2	2648.78	<0.001	60.11	<0.001
SS T1	1346.17	<0.001	18.70	<0.001
SS T2	1324.47	<0.001	25.54	<0.001
Anxiety T1	679.27	<0.001	20.65	<0.001
Anxiety T2	601.11	<0.001	25.01	<0.001
PF T1	77.18	<0.001	3.45	<0.001
PF T2	74.59	<0.001	6.48	<0.001
SEM	639.58	<0.001	2.64	0.008
Students with EP				
AS T1	2223.30	<0.001	38.98	<0.001
AS T2	2461.33	<0.001	49.60	<0.001
SS T1	1905.99	<0.001	22.22	<0.001
SS T2	1725.52	<0.001	33.70	<0.001
Anxiety T1	872.07	<0.001	27.37	<0.001
Anxiety T2	759.43	<0.001	28.99	<0.001
PF T1	94.70	<0.001	2.08	0.037
PF T2	66.57	<0.001	6.66	<0.001
SEM	1036.18	<0.001	7.49	<0.001

Abbreviations: AS, academic stress; EP, excellent performance; LDs, learning difficulties; PF, present focus; SS, self‐stigma.

In the current study, SEM was used to conduct cross‐lagged analysis, which enabled the exploration of possible temporal relationships between academic stress, self‐stigma, and anxiety level among students with LDs and students with excellent performance, respectively. In the present study, the total score of each scale was used as the observed variable indicator in the cross‐lag models of academic stress, self‐stigma, and anxiety level among students with LDs and among students with excellent performance. Evaluation criteria for CFA and SEM with good model fit were as follows: CFI > 0.90, TLI > 0.90, RMSEA < 0.08, SRMR < 0.08 [53]. Then, using the PROCESS macro in SPSS, we conducted regression analyses to test the moderating effect of present focus at T2 on the temporal link from academic stress at T1 to anxiety level at T2 among students with LDs and students with excellent performance, respectively. To determine whether the moderating effects were statistically significant, we used bias‐corrected bootstrap 95% confidence intervals based on 5000 bootstraps and employed Model 1 to investigate the moderating effects.

## 3. Results

### 3.1. Descriptive Data and Correlations

In the current study, the variables may be related to mental disorders. Therefore, in order to illustrate the participants’ psychiatric backgrounds, we compared the academic stress level of the participants in the present study with that of the senior high school students (*n* = 1349, M ± SD = 55.93 ± 9.86) in the study describing the Middle School Students Stressors Questionnaire development [55], and compared anxiety level with the norm of the Chinese version of STAI. The results (Table 3) showed that the academic stress of senior high school students (no matter the total sample, students with excellent performance, or students with LDs) in the current study was lower than that of senior high school students (*n* = 1349, M ± SD = 55.93 ± 9.86) in the study by Chen [55]. However, the anxiety level of senior high school students was higher than the anxiety level of the Chinese norm (M ± SD = 45.31 ± 11.99) of the STAI [64], but was significantly lower than the anxiety level of those who are diagnosed with depression (M ± SD = 57.22 ± 10.48) [58]. Table 4 presents correlations for academic stress, anxiety level, self‐stigma, and present focus across the two time points among the students with LDs and students with excellent performance. The results showed that significant positive correlations between academic stress, anxiety level, and self‐stigma were evident across the two waves. Besides, academic stress, anxiety level, and self‐stigma in the two periods were all significantly self‐correlated. Whereas, only present focus at T2 was negatively correlated with anxiety levels at T1 and T2.

**Table 3:** Comparison of academic stress and anxiety level of the participants in the present study with that of other populations.

Variables in the poplulation of present study vs in other population	Total sample (*n* = 2613)		Students with EP (*n* = 733)		Students with LDs (*n* = 735)	
	M ± SD	*t*	M ± SD	*t*	M ± SD	*t*
Academic stress T1 vs. other population’s academic stress	51.34 ± 9.36	−4.56 ^∗∗∗^	49.60 ± 10.02	−7.66 ^∗∗∗^	52.65 ± 8.53	−3.54 ^∗∗∗^
Academic stress T2 vs. other population’s academic stress	51.40 ± 9.73	−4.49 ^∗∗∗^	49.71 ± 10.09	−7.58 ^∗∗∗^	52.21 ± 9.32	−3.24 ^∗∗∗^
Anxiety T1 vs. norm	46.65 ± 10.42	6.57 ^∗∗∗^	45.76 ± 10.69	1.14	47.14 ± 9.96	4.87 ^∗∗∗^
Anxiety T2 vs. norm	47.52 ± 9.90	11.41 ^∗∗∗^	46.74 ± 10.21	3.79 ^∗∗∗^	47.80 ± 9.74	6.96 ^∗∗∗^
Anxiety T1 vs. depressed individuals’ anxiety level	46.65 ± 10.42	−11.50 ^∗∗∗^	45.76 ± 10.69	10.98 ^∗∗∗^	47.14 ± 9.96	−7.85 ^∗∗∗^
Anxiety T2 vs. depressed individuals’ anxiety level	47.52 ± 9.90	−7.62 ^∗∗∗^	46.74 ± 10.21	9.45 ^∗∗∗^	47.80 ± 9.74	−7.35 ^∗∗∗^

^∗∗∗^ *p*  < 0.0001.

**Table 4:** Correlations for the study variables among students with LDs and students with EP.

Variables	Students with LDs							Students with EP						
	1	2	3	4	5	6	7	1	2	3	4	5	6	7
1 AS T1	1.00	—	—	—	—	—	—	1.00	—	—	—	—	—	—
2 AS T2	0.42 ^∗∗∗^	1.00	—	—	—	—	—	0.48 ^∗∗∗^	1.00	—	—	—	—	—
3 A T1	0.30 ^∗∗∗^	0.24 ^∗∗∗^	1.00	—	—	—	—	0.36 ^∗∗∗^	0.29 ^∗∗∗^	1.00	—	—	—	—
4 A T2	0.16 ^∗∗∗^	0.32 ^∗∗∗^	0.41 ^∗∗∗^	1.00	—	—	—	0.25 ^∗∗∗^	0.40 ^∗∗∗^	0.40 ^∗∗∗^	1.00	—	—	—
5 SS T1	0.43 ^∗∗∗^	0.25 ^∗∗∗^	0.40 ^∗∗∗^	0.23 ^∗∗∗^	1.00	—	—	0.44 ^∗∗∗^	0.34 ^∗∗∗^	0.37 ^∗∗∗^	0.30 ^∗∗∗^	1.00	—	—
6 SS T2	0.25 ^∗∗∗^	0.41 ^∗∗∗^	0.26 ^∗∗∗^	0.43 ^∗∗∗^	0.47 ^∗∗∗^	1.00	—	0.32 ^∗∗∗^	0.50 ^∗∗∗^	0.26 ^∗∗∗^	0.43 ^∗∗∗^	0.55 ^∗∗∗^	1.00	—
7 PF T1	0.03	0.03	0.01	−0.01	0.01	−0.01	1.00	−0.09 ^∗^	−0.04	−0.04	−0.05	−0.12 ^∗∗^	−0.06	1.00
8 PF T2	0.05	0.03	−0.02	0.01	0.04	0.02	0.24 ^∗∗∗^	−0.05	0.04	−0.06	0.01	−0.07	−0.11 ^∗∗^	0.34 ^∗∗∗^

Abbreviations: A, anxiety; AS, academic stress; PF, present focus; SS, self‐stigma.

^∗^ *p* < 0.05

^∗∗^ *p* < 0.01

^∗∗∗^ *p* < 0.001.

### 3.2. Cross‐Lagged Analyses

To examine the reciprocal relationships between academic stress, anxiety level, and self‐stigma, SEMs with the total score of each scale as the observed variable indicator for academic stress, self‐stigma, and anxiety level at T1 and T2, and with demographic variables (i.e., gender, age, grade in the school, residency, and whether the only child in the family or not) as covariates were conducted. Consistent with Anderson and Gerbing’s [65] two‐step approach, we conducted CFA to evaluate the measurement model prior to full SEM testing. This included all latent constructs: academic stress, self‐stigma, and anxiety. Results demonstrated adequate convergent validity (composite reliability [CR] > 0.70 and average variance extracted [AVE] > 0.50 [acceptable if AVE > 0.40] [66]) and discriminant validity (square root of AVE > interconstruct correlations) [67] (Table 5).

**Table 5:** The convergent and discriminant validity of the constructs.

Variables	Students with LDs					Students with EP				
	CR	AVE	1	2	3	CR	AVE	1	2	3
1. Academic stress	0.81	0.59	**0.77**	—	—	0.89	0.73	**0.85**	—	—
2. Anxiety	0.88	0.45	0.37	**0.67**	—	0.89	0.48	0.40	**0.69**	—
3. Self‐stigma	0.94	0.83	0.54	0.46	**0.91**	0.90	0.76	0.53	0.43	**0.87**

*Note:* Diagonal = the square root of AVE; off‐diagonal = construct correlations. Bold values represent the square root of the average variance extracted (AVE), not correlation coefficients. These values are not related to statistical significance. According to discriminant validity criteria, when the square root of AVE for each construct exceeds the off‐diagonal inter‐construct correlation coefficients, it indicates good discriminant validity of the measurement model.

Abbreviations: EP, excellent performance; LDs, learning difficulties.

**Figure 1:**
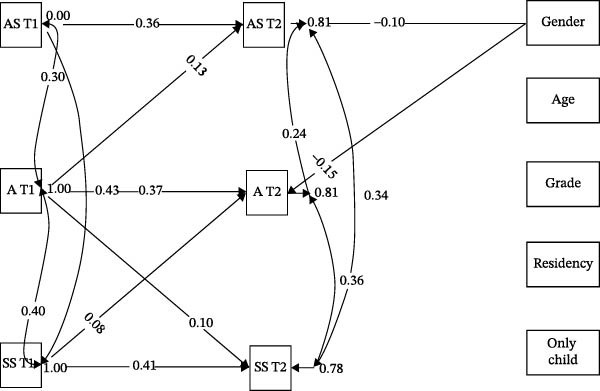
Structural equation model of academic stress, anxiety level, and self‐stigma among students with LDs. Only significant results were shown in the graph. AS, academic stress; A, anxiety; SS, self‐stigma.

The results of SEM showed that both of the models for students with LDs (*χ* ^2^/*df* = 2.64, *p* < .001, CFI = 0.96, TLI = 0.93, SRMR = 0.05, RMSEA = 0.05) and students with excellent performance (*χ* ^2^/*df* = 4.24, *p* < .001, CFI = 0.93, TLI = 0.88, SRMR = 0.08, RMSEA = 0.07) had good model fit indices. The results presented in model diagrams with standardized coefficients showed that among the students with LDs, academic stress at T1 only had a significantly positive relationship with academic stress at T2 (*β* = 0.36, *p* < 0.001), whereas self‐stigma at T1 had positively predictive links to self‐stigma (*β* = 0.42, *p* < 0.001) and anxiety level (*β* = 0.08, *p* = 0.031) at T2, and anxiety level at T1 had positively predictive links to anxiety level (*β* = 0.37, *p* < 0.001), academic stress (*β* = 0.13, *p* = 0.001), and self‐stigma (*β* = 0.10, *p* = 0.006) at T2 (Figure 1). The results indicated that for the students with LDs, academic stress, self‐stigma, and anxiety level are moderately stable over time. Furthermore, the results suggested that there was a one‐way temporal link from anxiety level to academic stress, while there was a two‐way temporal relationship between anxiety level and self‐stigma among students with LDs. In addition, the results showed that academic stress, self‐stigma, and anxiety level are correlated with each other at T1 and T2, respectively, which indicates that the correlation among the three variables is stable over time.

**Figure 2:**
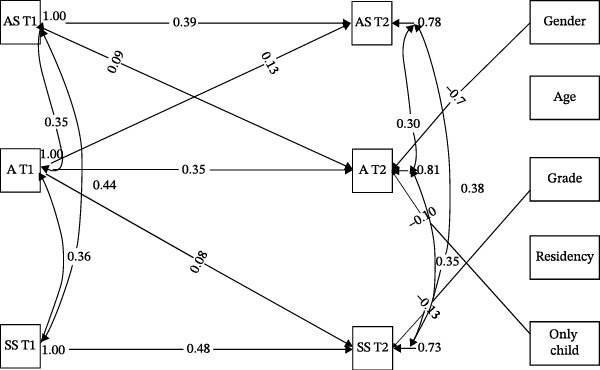
Structural equation model of academic stress, anxiety level, and self‐stigma among students with excellent performance. Only significant results were shown in the graph. AS, academic stress; A, anxiety; SS, self‐stigma.

Whereas among the students with excellent performance, academic stress at T1 was positively related to academic stress (*β* = 0.39, *p* < 0.001) and anxiety level (*β* = 0.09, *p* = 0.015) at T2, anxiety level at T1 was also positively linked to anxiety level (*β* = 0.35, *p* < 0.001), academic stress (*β* = 0.13, *p* < 0.001), and self‐stigma (*β* = 0.10, *p* = 0.026) at T2, whereas self‐stigma at T1 only had a reciprocal relationship with itself (*β* = 0.49, *p* < 0.001) at T2 (Figure 2). The results indicated that there is only a significant two‐way relationship between anxiety level and academic stress among students with excellent performance. In addition, the results showed that academic stress, self‐stigma, and anxiety level are correlated with each other at T1 and T2, respectively, which indicates that the correlation among the three variables is also stable over time.

### 3.3. Moderation Analyses

According to Hypothesis 3, present focus may moderate the effects of academic stress on anxiety level. The macro PROCESS (Model 1) was adopted to test the moderation models, in which academic stress at T1 or self‐stigma at T1 was included as the independent variable, present focus at T2 as the moderator, state anxiety present at T2 as the dependent variable, and the demographic variables (i.e., gender, age, grade in the school, residency, and whether the only child in the family or not) as covariates in students with LDs and students with excellent performance, respectively. The results showed that present focus only moderated the association between academic stress at T1 and anxiety level at T2 (*β* = 0.01, *t* = 2.07, *p* = 0.039, 95% CI [0.001, 0.024]). Specifically, high present focus at T2 exaggerated the temporal association of academic stress at T1 with anxiety level at T2 among students with LDs.

**Figure 3:**
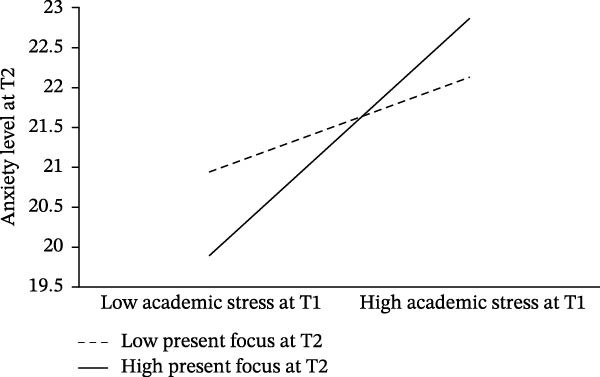
Moderation effect of present focus on the temporal relationship between academic stress and anxiety levels among students with LDs.

To better explain the moderation model, based on the average score of present focus plus or minus one standard deviation, the participants were divided into a high present focus group (M + SD) and a low present focus group (M − SD). Through simple slope analysis, we further examined the temporal links from academic stress at T1 to anxiety level at T2 under different levels of present focus. Results showed that the temporal links from academic stress at T1 to anxiety level at T2 among students with a high level of present focus (*β*_simple_ = 0.18, *t* = 4.89, *p* < 0.001) was stronger than that among students with low level of present focus (*β*_simple_ = 0.07, *t* = 1.80, *p* = 0.07) (Figure 3), suggesting that high present focus exaggerated the temporal association between academic stress at T1 and anxiety level among students with LDs.

## 4. Discussion

The present study investigated the reciprocal relationships between academic stress, self‐stigma, and anxiety level and the moderating effect of present focus on these associations among senior high school students with LDs and students with excellent performance, respectively, in China, a country with an academics‐focused culture. As had been hypothesized, academic stress, self‐stigma, and anxiety level were correlated with each other over time. Besides, the study revealed a one‐way temporal link from anxiety level to academic stress, while a two‐way temporal relationship between anxiety level and self‐stigma among students with LDs. Whereas there was only a temporally reciprocal association between anxiety level and academic stress among students with excellent performance. Furthermore, present focus moderated the predictive relationship between academic stress and anxiety level among students with LDs rather than among the students with excellent performance.

In the current study, correlation analysis results showed that academic stress, self‐stigma, and anxiety level were significantly intracorrelated and correlated with each other at T1 and T2, respectively, indicating that the state of the academic stress, self‐stigma, and anxiety level, and their relationships among the senior high school students with LDs and students with excellent performance were stable across time. The result was consistent with a recent longitudinal study showing the stress and anxiety are significantly intracorrelated and correlated with each other over time [42]. Another study also showed academic stress has been linked to a variety of negative effects, including ill health, anxiety, depression, and poor academic performance [41].

Results of cross‐lag analysis indicated there was a reciprocally temporal association between anxiety level and self‐stigma among students with LDs. The result was consistent with prior studies showing self‐stigma is positively associated with negative emotional reactions [20], higher level of psychological distress [17, 21], secondary traumatic stress [22], and anxiety among mothers whose children were diagnosed with ASD [25], and could predict depressive symptoms [23, 24]. According to the situational model by Corrigan and Watson [68], persons with a salient stigmatizing condition perceive that situation as the prerequisites for the occurrence of self‐stigma. Students with LDs are more likely to encounter conditions of poor academic performance, which are regarded as a disgrace in China, and thus are more likely to be stigmatized by their parents, educators, and peers, and perceive the stigmatizing situation, which might increase their self‐stigma and worries about being judged based on their academic performance. Studies showed that in Asian areas, low academic achievers are also likely to be targets of biased perceptions and to generate self‐stigma [3]. It is enlightening that we can reduce the level of self‐stigma by increasing the encouragement and recognition of students with LDs, and then adjust them to maintain an appropriate level of anxiety. An extensive training scheme that might help educators facilitate the coping strategies of students with LDs may preclude educators, peers, and parents from misunderstanding and stigmatizing these students, and subsequently lower the anxiety level [3].

Results of cross‐lag analysis also showed there was a reciprocally temporal association between anxiety level and academic stress among students with excellent performance. The finding was consistent with the finding that there was a longitudinal relationship between stress and anxiety [42]. Previous studies suggest that individuals experience mental and physiological changes when encountering stress. Specifically, the feelings of stress could lead to psychological disorders such as anxiety and depression [69].

Results also showed that present focus moderated the temporal link from academic stress at T1 to anxiety level at T2 among students with LDs rather than among students with excellent performance. Specifically, higher present focus level boosted the association of academic stress at T1 with the anxiety level at T2 among students with LDs. Whereas among students with excellent performance, present focus did not moderate the temporal link from academic stress at T1 to anxiety level at T2. In China, the students with excellent performance, who are viewed as one of the best students by teachers, parents, peers, and even themselves based on their current excellent academic situations, are less likely to be negatively influenced by focusing on their current excellent academic situations, thus the effect of academic stress is less likely to be moderated by the present focus among students with excellent performance. Whereas the students with LDs high in present focus may be more likely to perceive or focus on their current poor academic situations and stress, and thus exacerbating the effect of academic stress on anxiety level. The result was consistent with one prior study showing that students high on present temporal focus may be well aware of the risk situation and have the effects of temporal focus on emotions amplified [49]. Prior studies suggested that reactions to stress may vary depending on the individuals’ characteristics [35, 70]. Temporal focus often serves as a moderator of other relationships by either increasing or filtering out information [47]. Having a strong “here and now” orientation could lead individuals to rely more on present‐day experiences and immediate surroundings, perceiving such information as more salient for attitudes and behaviors [48]. A recent study showed that time perspective moderates the effect of bullying victimization (stress) on self‐esteem in adolescents [44]. Another empirical study showed that individuals with an anxiety disorder are more present fatalistic [71].

Researchers posited an inclination to focus on and emphasize one particular time frame may be critical in defining one’s vulnerability to psychological disorders since they function as cognitive temporal biases and distort the individual’s memory, attention, and information processing patterns [43, 72], and thus limit an individual’s ability to conform to situational demands [73]. For instance, individuals with strong present orientation are at greater risk of adverse outcomes such as mental health problems when they live in future‐oriented societies [74], and present‐fatalistic time perspective is associated with psychological distress, which is explained through people’s perception of their current lives as full of adversities with a minimal chance of improvement [75]. Besides, according to life history theory (LHT), an integrative theoretical framework that can illustrate how behavioral variation is contingent on experiences in the social‐developmental environment [76], as the product of an evolved psychological system designed to take advantage of fleeting opportunities, shorter time horizons, stronger present orientation, and weaker future orientation are associated with socially undesirable outcomes. Furthermore, individuals who are more present‐oriented may exhibit a form of hyper‐vigilance in assessing their environment and responding accordingly, which would be useful, since the unstable environments associated with shorter time horizons may change quickly and the threats may be more imminent [77].

According to the time perspective theory, present temporal perspective can be operationalized as present‐hedonistic and present‐fatalistic. Present‐hedonistic is characterized by an orientation toward present enjoyment, pleasure, and excitement, without sacrificing today for rewards of tomorrow, and relates to a hedonistic, risk‐taking, and pleasure‐oriented attitude toward life, with high impulsivity and little concern for future consequences of one’s actions. That is, present hedonistic students with LDs might have a vivid social life and put emphasis on enjoying their life; therefore—irrespectively to whether they are motivated—they may spend a significant amount of time with social events, parties, and other joyful activities, which allows less time spent with coping with the academic stress (i.e., the current academic situations), and can further lead to higher anxiety level.

Whereas present‐fatalistic reveals a belief that the future is predestined and uninfluenced by individual action, whereas the present must be borne with resignation because humans are at the whimsical mercy of fate [78]. In the current study, higher present‐fatalistic students with LDs might be more likely to pessimistically and fatalistically view the current circumstances, and they may perceive their current lives as full of adversities with a minimal chance of improvement [75], and believe that present behaviors have little impact on improving their future, which leads to a higher level of anxiety.

Besides, an overemphasis on past, present, or future could limit an individual’s ability to conform to situational demands, and may them function as cognitive temporal biases and distort the individual’s memory, attention, and information processing patterns, which might ultimately lead to negative outcomes [72, 73]. Furthermore, according to the LHT, shorter time horizons, stronger present orientation, and weaker future orientation may be the product of an evolved psychological system designed to take advantage of fleeting opportunities. Individuals who are more present‐oriented may exhibit a form of hyper‐vigilance in assessing their environment and responding accordingly [77], which would be more likely to induce the emotion of anxiety.

This is one of the few studies that investigated the relationship between academic stress, self‐stigma, and anxiety levels of Chinese senior high school students with LDs and with excellent performance who would attend the highly competitive college entrance examination (Gaokao) within 3 years. The result that the different reciprocal relationships among academic stress, self‐stigma, and anxiety levels in students with LDs and students with academic excellence might reflect the unique influence of Chinese culture and the educational system. In China, students’ academic performance is often regarded as an important indicator of personal value and family honor [79], and failure risks are thus magnified into “collective shame,” particularly in rural‐to‐urban migrant families where education is the only mobility channel in China. Besides, the “one exam determines one’s life” nature of Gaokao makes academic failure is regarded as “life collapse” [80]. Thus, students with LDs described struggles as “disgracing the family” and confronted with being filtered out before the battle, thus they are more likely to fall into the self‐labeling trap of “failure” which further induces anxiety, while high‐achievers are plagued by “status maintenance anxiety” because of confronting high expectations from surrounding people and the pursuit of academic success in the schools that are extremely competitive [81, 82].

The results are of great practical significance for the prevention and intervention of anxiety problems, enhancement of ability for school adaptation, and improvement of physical and mental development of high school students with LDs and students with excellent performance in China.

First of all, the current study revealed the different patterns of associations among academic stress, self‐stigma, and anxiety level in students with LDs and those with excellent performance. Specifically, for students with LDs, anxiety level was temporally linked to academic stress 1 month later, had a reciprocal relationship with self‐stigma, and present focus exacerbated the predictive association of academic stress with anxiety level. Whereas among students with excellent performance, anxiety level was temporally linked to self‐stigma, and had a reciprocal relationship with academic stress. This suggests that both personalized and common educational and psychological programs could facilitate decreasing the distress of students with LDs and students with excellent performance.

Specifically, the preventions and interventions for solving psychological distress for students with LDs or excellent performance could include school policy, psychoeducation, and counseling. Specifically, the school policymakers were suggested to create a more inclusive learning and educational environment, such as abolishing public academic ranking, and equipping educators to spot early warning signs and provide differentiated mental support. Besides, schools are supposed to ensure accessible counseling pathways, conduct mental health assessment, and set up workshops on anxiety management regularly.

Regarding the psychoeducation, parents are suggested to cultivate reasonable expectations for academic achievement of their children and to realize that achievement is separate from worth, teachers are suggested to recognize varied psychological profiles of students, and students with LDs are suggested to learn practical self‐advocacy skills and to be trained to challenge negative self‐beliefs, and students with excellent performance are educated to focus on healthy achievement mindsets, recognize and manage perfectionism, understand the value of mistakes and setbacks, develop stress‐reduction techniques, and foster self‐compassion.

As for the counseling, cognitive behavioral therapy (CBT) and group CBT, targeted at self‐stigma, stress, and anxiety, are highly recommended. Key elements of the therapy for students with LDs could include identifying and restructuring maladaptive thoughts (e.g., I will never succeed), developing coping strategies for academic frustration, and building social skills to reduce isolation. Besides, the programs aimed to reduce present focus could decrease the predictive association from academic stress to anxiety level among students with LDs. The counseling support focusing on managing performance anxiety, fear of failure, setting realistic expectations, and navigating social pressures related to achievement is recommended, and promoting help‐seeking behaviors is highlighted for students with excellent performance.

What is more, since there is a bidirectional association between anxiety level and self‐stigma among students with LDs, and between anxiety level and academic stress among students with excellent performance. The findings imply that educational programs need to address both mental health and self‐perception issues simultaneously, as improvements in one area can positively influence the other. That is, the prevention and intervention that target reducing anxiety level could also benefit from the prevention and interventions that target reducing academic stress and self‐stigma. Besides, the study’s cross‐lag analysis over a 5‐week period provides insights into the timing of interventions. Thus, school counselors can focus on and identify students experiencing academic stress and provide timely interventions, which is essential for the early identification and support of students with mental health issues.

But there are several limitations of the current study that should be considered. First, the participants were recruited using convenience sampling and only from two key senior high schools and three general senior high schools in Liaoning Province in China with an Asian culture that highly values academic excellence, which indicates the low representativeness of our study sample, thus restricting the generalizability of our study results. Studies with participants from different areas and cultures are needed to corroborate our findings. Besides, the study did not collect socioeconomic status (SES) data from participants, which limits the ability to examine the potential influence of SES on self‐stigma, academic stress, and anxiety levels and may affect the generalizability of findings across different socioeconomic groups. Future research should also incorporate SES as a key variable. Second, only self‐report measures were used. Future research may use additional data sources, such as the family members, teachers, and peers of the participants. Third, the interval between data collection at T1 and T2 was 5 weeks, and the reciprocally predictive relationship between academic stress, self‐stigma, and anxiety level over longer period needs to be further studied in the future.

## 5. Conclusions

This study revealed the temporal associations between academic stress, self‐stigma, and anxiety level among Chinese senior high school students with LDs are different from those among students with excellent performance. The results revealed a one‐way link from anxiety level to academic stress, while a two‐way effect between anxiety level and self‐stigma among students with LDs. Whereas there was only a significant reciprocal relationship between anxiety level and academic stress among students with excellent performance. Moreover, higher present focus exaggerated the temporal link from academic stress to anxiety level among students with LDs. The results hold implications for the prevention and intervention targeting the reduction of anxiety among students with LDs and students with excellent performance.

## Funding

This work was supported by the Research Institute in Humanities and Social Sciences of the Ministry of Education (Grant 23YJC190034), the Research Project of Henan Federation of Social Sciences (Grant SKL‐2025‐933), the Scientific Research Innovation project of China University of Political Science and Law (Grant 23ZFG18001), the Fundamental Research Funds for the Central Universities, the Philosophy and Social Sciences Planning Project of Henan Province of China (Grant 2025CJY056), and the 2025 Zhengzhou University College Students’ Innovation Training Program (Grant 202510459037).

## Disclosure

The funding sources had no involvement in study design, in the collection, analysis and interpretation of data, in the writing of the report, and in the decision to submit the article for publication.

## Conflicts of Interest

The authors declare no conflicts of interest.

## Data Availability

The datasets generated during and/or analyzed during the current study are available from the corresponding author upon reasonable request.
